# Synaptic mitochondrial respiration differs between the prefrontal cortex and hippocampus in female, but not male, mice: no effect of chronic stress history

**DOI:** 10.21203/rs.3.rs-7643085/v1

**Published:** 2025-10-15

**Authors:** Gladys A. Shaw, Amy J. Wegener, Hannah Stadtler, Gretchen N. Neigh

**Affiliations:** Virginia Commonwealth University

**Keywords:** mitochondria, stress, estrogen, anxiety

## Abstract

We evaluated the impact of chronic repeated predation stress (CRPS) on presentation of anxiety-like behavior and synaptic mitochondrial respiration within the prefrontal cortex (PFC) and hippocampus (HPC). Male and female C57Bl/6NTac mice were subject to CRPS for 15 days during their adolescent (PND36–50) and early adult (PND57–71) stages. All animals were assessed for anxiety-like behavior in the open-field assay in adulthood. Brains were collected (PND106–108) and immediately used to assess synaptic mitochondrial respiration with the SeahorseXFe24 instrument. CRPS induced anxiety-like behavior in both male and female mice in the open field, despite not observing stress effects on mitochondrial respiration within either sex. However, females displayed significant region-specific differences that were not reflected within the males. PFC mitochondria respiration was higher the synaptic mitochondrial respiration rates in the HPC across all mitochondrial dynamics in female, but not male, mice. To further understand regional differences in mitochondrial respiration, we analyzed expression of ESR2 and UCP2 as both are indicated in regulation of mitochondrial dynamics. In the HPC, females expressed higher levels of both genes compared to males which may contribute to regional differences in females. Further, hippocampal ESR2 expression was elevated by CRPS in both sexes, suggesting a potential mechanism by which synaptic mitochondria are protected.

## 1. Introduction

Rates of depression and anxiety in America have increased significantly in the past decade, with the first age of onset younger than it has been in previous generations (Kessler et al., 2005; Merikangas et al., 2010). There is an increased incidence of these mood disorders in females over males, with a rapid uptick of diagnoses in females occurring in the teenage years (Altemus et al., 2014). However, many studies only include male subjects, discordant with the established sex difference in the incidence of mood disorders (Pacholko et al., 2019; Tenkorang et al., 2018).

The prefrontal cortex (PFC) and hippocampus (HPC) are primary regions of interest in mood disorders as both are regulatory regions in the modulation of the hypothalamic-pituitary-adrenal axis activity. The role of early life stress on mitochondrial function at the synapse has been demonstrated with maternal separation altering mitochondria and synaptic maturity in both males and females (Wei et al., 2015), as well as data from our group suggesting changes in hippocampal synaptic composition following chronic psychosocial stress in female Wistar rats (Hyer et al., 2021). As studies have suggested that mitochondria within the synapse exhibit the first signs of cellular dysfunction (Marazziti et al., 2012; Olesen et al., 2020; Song et al., 2021), our focus will be on synaptic mitochondria within the PFC and HPC.

Recently, our group has shown that chronic steady state exogenous corticosterone (CORT) administration in adult C57Bl/6 mice yields a treatment specific shift in mitochondrial respiration as measured from HPC synaptosomes (Shaw et al., 2024). Yet, the long-lasting effects of an increase in basal CORT following an endogenous stressor in adolescence, as well as the mechanisms involved, on mitochondrial respiration within the PFC and HPC is still unknown. These data uncover the role of chronic stress in the induction of altered transcript and protein level expression of ERβ within synaptic mitochondria, potentially mediating sex- and stress-differences in synaptic mitochondrial respiration within the PFC and HPC.

## 2. Methods

### 2.1 Animals

Forty postnatal day (PND) 22 male and female C57Bl/6 mice were purchased from Taconic Laboratories. All mice were placed in same sex pairs and given a single nestlet square for enrichment. The temperature and humidity-controlled colony room was kept on a 12:12 light:dark cycle, with *ad libitum* access to both food and water in ventilated rack cages. Protocols used were approved by Virginia Commonwealth University’s Animal Care and Use committee.

### 2.2 Chronic Repeated Predation Stress (CRPS)

Beginning on PND 36, mice were randomly assigned to undergo CRPS and were isolate housed with no enrichment. During CRPS sessions, mice were placed in a clean dwarf hamster ball to provide protection from physical harm. The ball was placed in the home cage of an adult male Long Evans rat for 30 minutes daily for two weeks during adolescence (PND 36–50) and during adulthood (PND 57–71) (Shaw, Bent, et al., 2020; Shaw, Hyer, et al., 2020).

### 2.3 Open Field

As a test for anxiety-like behavior, all mice were subject to 10 minutes in a grey open field arena (36cm × 36cm × 43cm) under 700 lux lighting three to five hours into the light cycle. Movement was tracked in real time using EthoVision XT 15.0 and an overhead camera at 25 frames per second. Center-point of the animal was used to assess velocity, distance traveled, and location in the arena.

### 2.4 Tissue Collection

Animals were euthanized via rapid decapitation. Mice were not placed under anesthesia, as anesthesia alters corticosterone levels in rodents (Bekhbat et al., 2016). Fresh tissue was collected at PND 106–108. The PFC and HPC from the right hemisphere were fresh dissected and immediately processed for synaptosomal isolation. PFC and HPC tissue from the left hemisphere were dissected and flash frozen for qPCR analysis, as both ERβ and UCP2 are transcribed in the nucleus.

### 2.5 Synaptosomal Mitochondria Isolation

Tissue was processed according to previous publications (Dunkley et al., 2008; Shaw et al., 2021; Shaw, Hyer, et al., 2020). Using a 2mL Dounce homogenizer, neural tissue was homogenized in 1.5mL homogenizing buffer using 12 strokes of the plunger. Homogenate was spun at 3600×g for 10 minutes at 4°C. 1.2mL of supernatant was then gently added atop a 5-layer discontinuous, isotonic Percoll gradient composed of 1mL of 23%, 15%, 10%, 3% Percoll, and 0.5 mL of 0% Percoll. Prepared gradients with homogenate were then spun at 32,000×g for 10 min at 4°C in a Sorvall MX-120 + ultracentrifuge and a fixed-angle S58A rotor.

Synaptosomes were removed from the interface of the 10% to 15% Percoll layers and the 15% to 23% Percoll layers and placed in a clean ultracentrifuge tube with 6mL of ionic media (isotonic solution; see (Shaw, Hyer, et al., 2020) for composition) and spun at 15,000×g for 35 minutes at 4°C in a fixed angle S58A rotor. Synaptosomes, pelleted at the bottom of the tube, were collected and assessed for protein content via absorbance measurements via NanoDrop.

### 2.6 Seahorse Cell Mito Stress Test

Isolated synaptosomes were diluted to 40μg synaptosomes per 100μL of ionic media and plated in triplicate in poly-D lysine coated Seahorse XFe24 cell plates (Agilent Technologies). The prepared plates were spun at 3400×g for 30 min at 4°C to allow adherence of synaptosomes. Plates were incubated in a non-CO_2_ incubator for 30 minutes before changing the ionic media for warmed Seahorse XF media. The cell mitochondrial stress test (Agilent Technologies, PN: 103015-100) was used to assess mitochondrial respiration, composed of Oligomycin A (2.0μM), FCCP (1.0μM), and Rotenone/Antimycin A (0.5μM).

### 2.7 RNA Extractions

Tissue was homogenized in 400μL RLT buffer via the Tissue Lyser II instrument (Qiagen) using 1.4mm ceramic beads (Omni International Cat # 19-645-3 Batch 2156390). Tissues were homogenized at 30/s for 45s (HPC) or 3min (PFC), followed by a 3 minute spin through a QiaShreader. All RNA extractions were completed using the Qiagen Mini Kit and QiaCube instrument. RNA concentration and purity were assessed via Nanodrop (ThermoFisher). RNA was diluted to 0.25μg/20μL for cDNA conversion and converted using the AB High-Capacity cDNA reverse transcriptase kit according to manufacturer’s instructions.

### 2.8 Quantitative Polymerase Chain Reaction

TaqMan quantitative polymerase chain reaction (qPCR) was completed on cDNA samples run in triplicate on a QuantStudio 6 instrument. Relative transcript levels of estrogen receptor ß (ESR2; ThermoFisher Scientific, Cat # Mm00599821_m1) and the mitochondrial uncoupling protein 2 (UCP2, ThermoFisher Scientific, Cat # Mm00627599_m1) were assessed. GAPDH (Cat. # Mm99999915_g1), and ß-actin (Cat. # Mm01205647_g1) were used for normalization of each sample as housekeeping genes. The non-stressed males were set as the control group for relative expression.

### 2.9 Statistical Analysis

Data from all behavioral and molecular tests was analyzed using GraphPad Prism 9.2 for Windows (GraphPad La Jolla, CA). An α = 0.05 was used for all statistical assessments.

## 3. Results

### 3.1 Open Field

**Figure 1:**
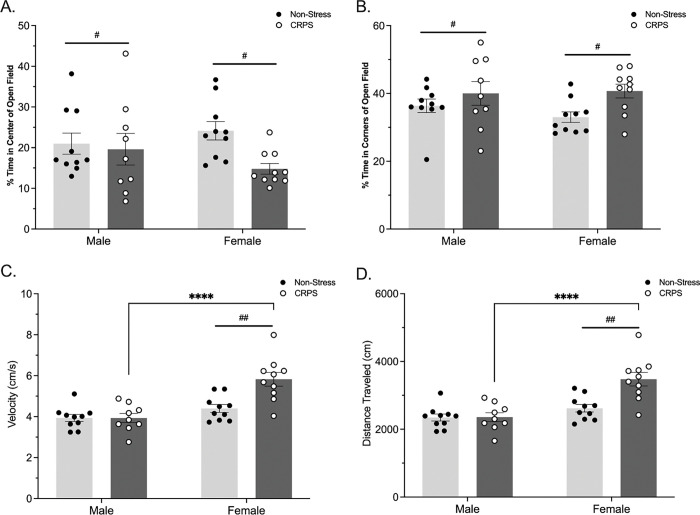
A history of CRPS induces anxiety-like behavior within the open field assay. A) Mice with a history of stress display increased anxiety-like behavior as shown by decreased time spent in the center of the arena. B) CRPS mice spent more time in the corners of the arena. In both C) velocity and D) distance traveled, females were more hyperactive than males. Females with a history of stress exhibited increased hyperactivity compared to non-stressed females and CRPS males. Bars represent mean ± SEM, #p<0.05 of overall effect of stress, ##p < 0.01 effect of stress, ****p < 0.0001 interaction effect.

A history of stress induced anxiety-like behavior within the open field. Stressed males and females spent less time in the center of the arena (F_(1,35)_ = 4.246, p = 0.0468; Fig. 1A) and more time in the corners of the open field arena (F_(1,35)_ = 6.004, p = 0.0194; Fig. 1B). The locomotor metric of velocity differed by sex (F_(1,35)_ = 24.06, p < 0.0001), stress (F_(1,35)_ = 8.753, p = 0.0055) and an interaction (F_(1,35)_ = 8.810, p = 0.0054; Fig. 1C), such that females and CRPS mice displayed hyperactivity. A post hoc Fisher’s LSD revealed CRPS females displayed a higher velocity compared to non-stressed females (p = 0.0002) and CRPS males (p < 0.0001). Distance traveled was impacted by sex (F_(1,35)_ = 23.91, p < 0.0001), stress (F_(1,35)_ = 9.312, p = 0.0043), and an interaction between the variables (F_(1,35)_ = 8.782, p = 0.0054; Fig. 1D). Again, a post hoc Fisher’s LSD indicated that CRPS females traveled further than non-stressed females (p = 0.0001) and CRPS males (p < 0.0001).

### 3.2 Synaptosomal Respiration

**Figure 2:**
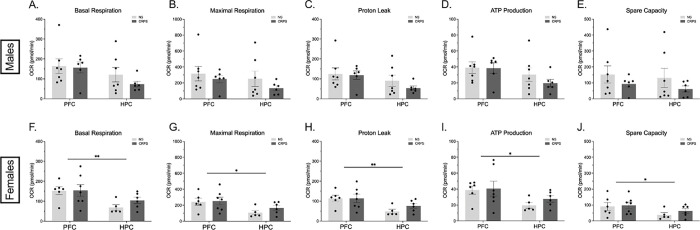
Chronic repeated predation stress does not impact synaptic mitochondrial respiration in the PFC or HPC. A-E) There were no impacts of CRPS or regional differences observed in synaptic mitochondrial respiration in males. (F-J) In females, there was no effect of CRPS on mitochondrial respiration, but HPC exhibited lower respiration compared to the PFC across all mitochondrial dynamics. Bars represent mean ± SEM, *p<0.05 effect of region, **p < 0.01 effect of region.

Due to persistent sex differences in metabolic data, all respiration metrics were analyzed within sex. In males, neither CRPS nor region impacted basal respiration (p > 0.05; Fig. 2A), maximal respiration (p > 0.05; Fig. 2B), proton leak (p > 0.05; Fig. 2C), ATP production (p > 0.05; Fig. 2D), or spare capacity (p > 0.05; Fig. 2E).

In females, assessment of mitochondrial dynamics shows that although CRPS had no statistical impact on OCR (p > 0.05), there are persistent region-specific differences. The PFC exhibited higher OCR in measures of basal respiration (F_(1,20)_ = 8.898, p = 0.0074; Fig. 2F), maximal respiration (F_(1,20)_ = 7.434, p = 0.0130; Fig. 2G), proton leak (F_(1,20)_ = 9.808, p = 0.0053; Fig. 2H), ATP production (F_(1,20)_ = 5.962, p = 0.0240; Fig. 2I), and spare capacity (F_(1,20)_ = 4.920, p = 0.0383; Fig. 2J).

### 3.3 qRT-PCR

**Figure 3:**
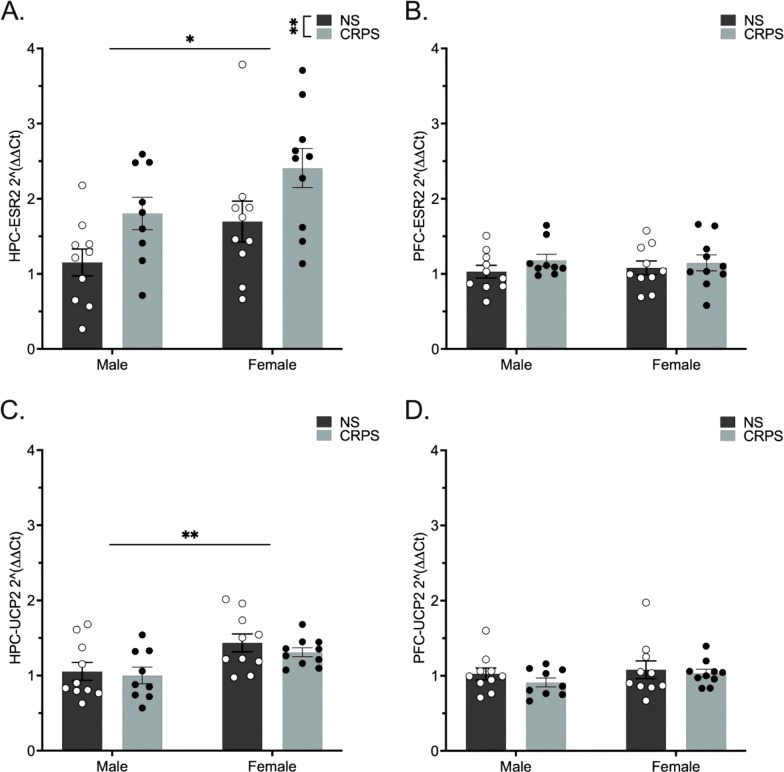
HPC ESR2 gene expression is impacted by sex and stress. HPC UCP2 gene expression is impacted by sex. A) Females have higher HPC ESR2 gene expression overall. HPC ESR2 gene expression is increased in both males and females following a history of CRPS. B)There are no sex or stress-induced differences in ESR2 expression in the PFC. C) Females express higher HPC UCP2 gene expression overall. D) UCP2 expression in the PFC is not impacted by sex or stress. Bars represent mean ± SEM, *p<0.05, **p < 0.01.

Transcript levels of ESR2, the gene transcribing ERβ, was assessed using a 2-way ANOVA for the factors sex and stress. A history of CRPS increased ESR2 hippocampal expression in both males and females (F_(1,35)_ = 8.324, p = 0.0067; Fig. 3A). Sex impacted hippocampal ESR2 expression such that females expressed higher levels of ESR2 compared to males (F_(1,35)_ = 5.922, p = 0.0202). UCP2, the gene transcribing the mitochondrial uncoupling protein UCP2, was not impacted by CRPS in the hippocampus (F_(1,35)_ = 0.7412, p > 0.05; Fig. 3C), but was higher in females regardless of stress history (F_(1,35)_ = 10.79, p = 0.0023). Expression of ESR2 in the PFC was not altered by stress (F_(1,35)_ = 1.420, p > 0.05; Fig. 3B) or sex (F_(1,35)_ = 0.007, p > 0.05). Similarly, UCP2 in the PFC was not altered by stress (F_(1,35)_ = 0.915, p > 0.05; Fig. 3D) or sex (F_(1,35)_ = 1.164, p > 0.05).

## 4. Discussion

The HPC and PFC are implicated in stress disorders, but likely exhibit distinct metabolic consequences following stress based on regional energetic requirements and resource availability for baseline function. Despite CRPS inducing anxiety-like behavior, we did not observe the predicted consequences of CRPS on mitochondrial respiration. Sex differences in regional variations of metabolic activity may be indicative of regional metabolic capacity for adapting to severe external stressors (Lee et al., 2013). In females, the PFC exhibited increased synaptic mitochondrial respiration compared to the HPC across all dynamics. As this regional difference was not observed in males, we sought to determine potential mechanisms that differ within regions between the sexes. We investigated expression of two genes that contribute metabolic output and may drive the observed regional differences.

The regional differences in mitochondrial dynamics observed in females suggests differing substrate level availability or necessity, likely supported by differences in substrate pools or due to a direct modulator of the electron transport chain (ETC). A previous mouse study determined that the HPC and cortex exhibit regional differences in substrate pool availability under basal conditions; however, this study did not specify the sex of the mice analyzed (Lee et al., 2013). Estrogens are known regulators of metabolic pathways (Chen et al., 2009; Nilsen et al., 2007). In order to tease apart the observed regional sex differences in the current study, we examined expression of the ESR2 and UCP2 genes that have established roles in modulating metabolic activity. We observed elevated expression of both genes in the HPC of female mice, relative to males, an effect not observed in the PFC. ATPase activity is directly modulated through ERβ (Álvarez-Delgado et al., 2010; Chen et al., 2009; Del Río et al., 2018; Irwin et al., 2008; Liao et al., 2019), acting as an activator of ATP production *in vitro* (Liao et al., 2019). Additionally, ERβ was elevated in both males and females in the HPC following CRPS. Given the neuroprotective nature of estrogen, we postulate that ESR2 expression is elevated in the CRPS animals to protect against the negative impacts of stress that have been reported in previous mitochondrial studies.

UCP2 acts to uncouple ATP production from the influx of protons between the inner membrane space and matrix of the mitochondria. This is important as it acts to increase the survival of neurons during periods of physiological stress (Normoyle et al., 2015). Moreover, UCP2 expression is neuroprotective as it decreases reactive oxygen species production and Ca^2+^ influx within mitochondria, preventing activation of an apoptotic cascade (Castora, 2019; Normoyle et al., 2015; Park et al., 2011). As such, in the HPC, females may benefit from elevated expression of both genes during basal function.

We have previously demonstrated that chronic exogenous corticosterone lowers synaptic mitochondrial respiration in the hippocampus of both male and female mice (Shaw et al., 2024). We postulated that a CRPS paradigm would illicit similar impacts on synaptic mitochondria, but these effects were not observed in the current study. Directionality of stress modulation on synaptic mitochondria respiration may be attributed to the type of stressor. Other studies have demonstrated chronic mild stress triggered mitochondrial respiratory inefficiency in cortical and hippocampal mitochondria not specific to the synapse (Gong et al., 2011). Similarly, mitochondria respiration in prefrontal punches is lowered in male rats following exposure to predator odor, but not females (Grigoruţă et al., 2020). However, chronic restraint stress elevates complex I-III activity in both male and female rats in the hippocampus (de Souza Mota et al., 2017). These studies support the role of mitochondria in preclinical stress paradigms. As such, the lack of effect of CRPS on synaptic respiration does not indicate that synaptic mitochondria in the current study were unresponsive to the paradigm, but perhaps exhibit adaptations reflected by other mitochondrial assessments.

Despite not observing an impact of CRPS on mitochondrial respiration in the current study, it could be beneficial to assess changes in morphological phenotype of these synaptic mitochondria. Previous data has shown that CRPS can influence synaptic mitochondrial morphology as indicated by the Flameng scoring system of mitochondrial health (Flameng et al., 1980; Shaw, Hyer, et al., 2020). In the aforementioned study, CRPS females had more synaptic mitochondria exhibiting a score of “4” indicating agranular and significantly damaged mitochondria. Such scorings suggest that CRPS females displayed more damaged mitochondria at the time of isolation while males did not have a visible change in mitochondrial morphology in response to stress. Further, this outcome was not reflected in CRPS female mitochondria respiration data. As such, the absence of synaptic respiration shifts does not guarantee mitochondria were not responsive to the stressor and requires future studies to fully characterize mitochondria morphology.

The increased presentation of anxiety-like behavior in the open field indicates that the CRPS paradigm was sufficient to alter behavior in both sexes. There was also an upregulation of ESR2 expression in the hippocampus following CRPS that may have elicited protective qualities in the hippocampus across CRPS animals that prevented a stress-induced shift in synaptic mitochondria respiration. Given the open field is not a hippocampal associated task, additional behavioral studies that evaluate performance on hippocampal associated memory tasks following CRPS and an estrogen receptor targeted intervention may provide insight on the combined impact of ESR2 and CRPS within this model.

## Supplementary Material

Supplementary Files

This is a list of supplementary files associated with this preprint. Click to download.

• floatimage1.png

## Data Availability

The datasets generated during the current study are not publicly available but are available from the corresponding author on reasonable request.
